# Molecular Targets of Pinocembrin Underlying Its Regenerative Activities in Human Keratinocytes

**DOI:** 10.3390/ph15080954

**Published:** 2022-07-31

**Authors:** Jirapak Ruttanapattanakul, Nitwara Wikan, Saranyapin Potikanond, Wutigri Nimlamool

**Affiliations:** 1 Department of Pharmacology, Faculty of Medicine, Chiang Mai University, Chiang Mai 50200, Thailand; jirapak.ken@gmail.com (J.R.); nitwara.wik@cmu.ac.th (N.W.); saranyapin.p@cmu.ac.th (S.P.); 2 Graduate School, Chiang Mai University, Chiang Mai 50200, Thailand

**Keywords:** flavonoids, pinocembrin, wound healing, keratinocyte, regenerative medicine

## Abstract

Pinocembrin is one of the well-known compounds in the group of flavonoids. The pharmacological activities of pinocembrin in association with wound-healing activities have been reported. However, its effects on the aspect of cellular interaction underlying growth and survival are still unidentified in human keratinocytes. Our previous study reported that *Boesenbergia rotunda* potently stimulated survival and proliferation of a human keratinocyte cell line (HaCaT). On the basis that pinocembrin is revealed to be one of the major constituents of this plant, we aimed to define the survival- and proliferation-enhancing effects of this compound at the cellular level. Results from the current study confirmed that pinocembrin induced an increase in HaCaT cell number. At the signaling perspective, we identified that pinocembrin significantly triggered ERK1/2 and Akt activation. The stimulating effects of pinocembrin were clearly inhibited by MEK and PI3K inhibitors authenticating that proliferation- and survival-promoting activities of pinocembrin were mainly acted on these two signaling cascades. Altogether, we successfully identified that pinocembrin functions to induce keratinocyte proliferation and survival, at least by provoking MAPK and PI3K pathways. Our study encourages the fact that pinocembrin is one of the interesting natural flavonoid compounds to be developed as a wound closure-promoting agent.

## 1. Introduction

Wound healing is an indispensable protective mechanism for both humans and animals. It consists of different crucial steps controlled by different stages composed of coagulation/hemostasis stage, inflammation stage, proliferation stage, and wound remodeling stage [1,2]. These phases are not individual but partly overlap on a sequence by hemostasis, inflammation, proliferation, and remodeling phase, respectively [3]. After a wound has occurred, coagulation cascade and platelet are activated by injured endothelial cells, and the next event is the generation of acute inflammation of the surrounding tissues for eliminating the pathogen [4]. Importantly, the proliferation step truly requires proliferation of surrounding cells which are activated by many growth factors including vascular endothelial growth factor (VEGF), platelet-derived growth factor (PDGF), and epidermal growth factor (EGF). The final phase called “wound remodeling period” is the longest phase which focuses on organization, degradation, and synthesis of the extracellular matrix in the dermis layer of the skin [5,6,7]. Natural delays in healing processes may occur in older individuals [8]. All phases of the healing process are affected, making cell proliferation, remodeling, and collagen synthesis to occur at a lesser degree. Moreover, in comparison to children and young adults, the elderly may have complications from certain diseases that greatly hamper the healing process [9]. Therefore, besides recently available healing therapies, discovery of novel active agents and special treatments that can effectively accelerate wound healing to the maximal level would be beneficial for the elderly to prevent possible complications such as septicemia. Considering this fact, natural compounds including flavonoids have emerged as interesting agents for enhancing wound healing.

Pinocembrin (PC) is one of the primary flavonoid compounds that is found in many plants belonging to different families, as *Boesenbergia rotunda* (Zingiberaceae) [10,11], and it has been previously reported that pinocembrin has many pharmacological effects including anti-inflammatory and anti-microbial effects, anti-aging activities, and wound-healing properties [12,13,14,15,16]. Regarding the wound-healing properties, our previous research found that *Boesenbergia rotunda* (BR), which is a plant in Zingiberaceae family [17], can promote keratinocyte cell proliferation via activating MAPK and PI3K pathways [18]. Additionally, it was found that *Boesenbergia rotunda* ethanolic extract contains pinocembrin as one of its major compounds [19]. Possibly, pinocembrin may be the responsible constituent of BR that stimulates proliferation of human keratinocytes. However, the wound healing-promoting activities of pinocembrin in keratinocytes has not been fully elucidated; especially in the view of specific activation of growth and survival pathways. On this basis, the major aims of this research are to determine the function of PC on keratinocyte proliferation and to monitor the expression and activation status of important kinase markers in the MAPK signal transduction pathway (ERK 1/2, pERK1/2) and PI3K/Akt signal transduction pathway (pAkt and Akt) in response to PC treatment.

Our study provided an insight into how an active compound, pinocembrin, functions at the cellular level for enhancing proliferation and survival which are crucial events for wound healing. This new understanding may guide for the possibility to develop this compound as an alternative wound healing-accelerating drug, especially for patients who have complications of diseases and are irresponsive to currently available standard treatments.

## 2. Results

### 2.1. Pinocembrin Affects the Viability of Human Keratinocytes

**Figure 1:**
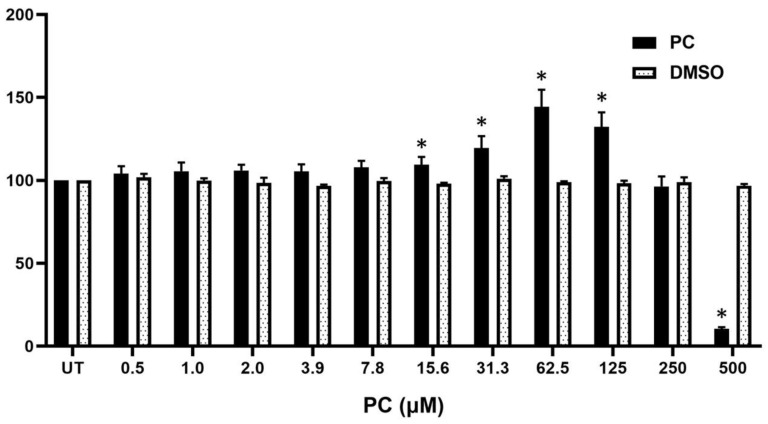
Cytotoxicity effects of pinocembrin on HaCaT cells determined by MTT assay. Human keratinocytes treated with different doses of pinocembrin for 48 h in serum-free media. Data were shown as percent cell viability/proliferation with mean ± SD. * *p* < 0.05 as compared with the un-treated group (UT).

To investigate proliferation-enhancing effects of pinocembrin, we first performed cytotoxicity of pinocembrin in HaCaT cells which are immortalized human keratinocytes. Results demonstrated that 500 μM was the only concentration of pinocembrin which significantly reduced the viability of HaCaT cells. Interestingly, pinocembrin at the range be-tween 15.6 and 125 μM presented a dramatic increase in cell viability/cell proliferation, with a maximal peak at 62.5 μM where the cell viability of HaCaT cells was approximately 140% in comparison to that of the untreated cells (100%) (Figure 1). Human keratinocytes treated with DMSO (as a vehicle control) at all concentrations did not show any change in cell viability.

### 2.2. Effects of Pinocembrin on Accelerating Scratch Wound Closure of Human Keratinocyte Monolayer 

**Figure 2:**
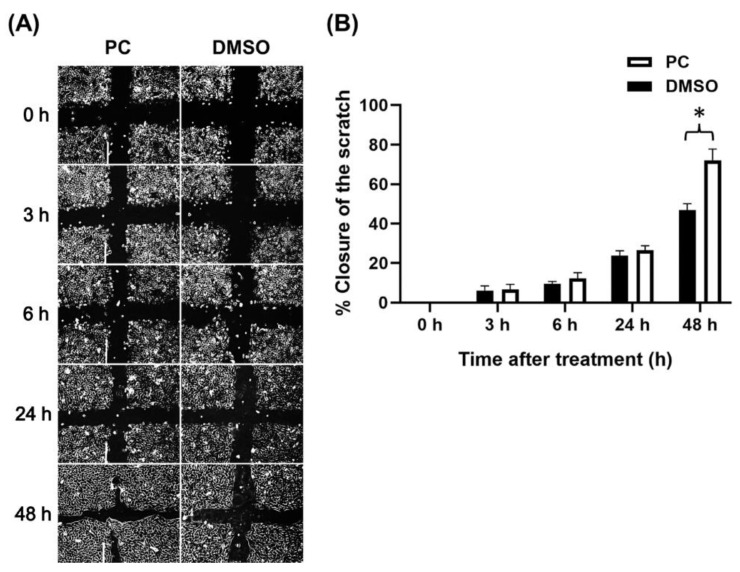
Effects of pinocembrin on accelerating closure of scratch wound of human keratinocyte monolayers. (**A**) Phase-contrast microscopy (10× magnification) of scratch wound-healing assay at various captured times (0 h, 3 h, 6 h, 24 h, and 48 h) in HaCaT treated with 62.5 μM pinocembrin; (**B**) Percent closure of the scratch wounded areas of human keratinocyte monolayer over the course of 48 h. Data were analyzed from three individual replicates and presented as mean ± SD. * *p* < 0.05 as compared with the DMSO control.

From the MTT results demonstrating that pinocembrin from 15.6 to 62.5 μM could increase the viability of human keratinocytes, it is reasonable that this increased cell viability may also promote healing of keratinocyte monolayer. Therefore, we evaluated wound closure-accelerating effects of pinocembrin on human keratinocyte monolayer by choosing the highest concentration at 62.5 μM. Phase-contrast micrographs demonstrated that pinocembrin significantly accelerated the cellular wound healing over time (0, 3, 6, 24, and 48 h), compared to the DMSO vehicle control group (Figure 2).

### 2.3. Pinocembrin Induces Proliferation and Increases the Size of HaCaT Colonies

**Figure 3:**
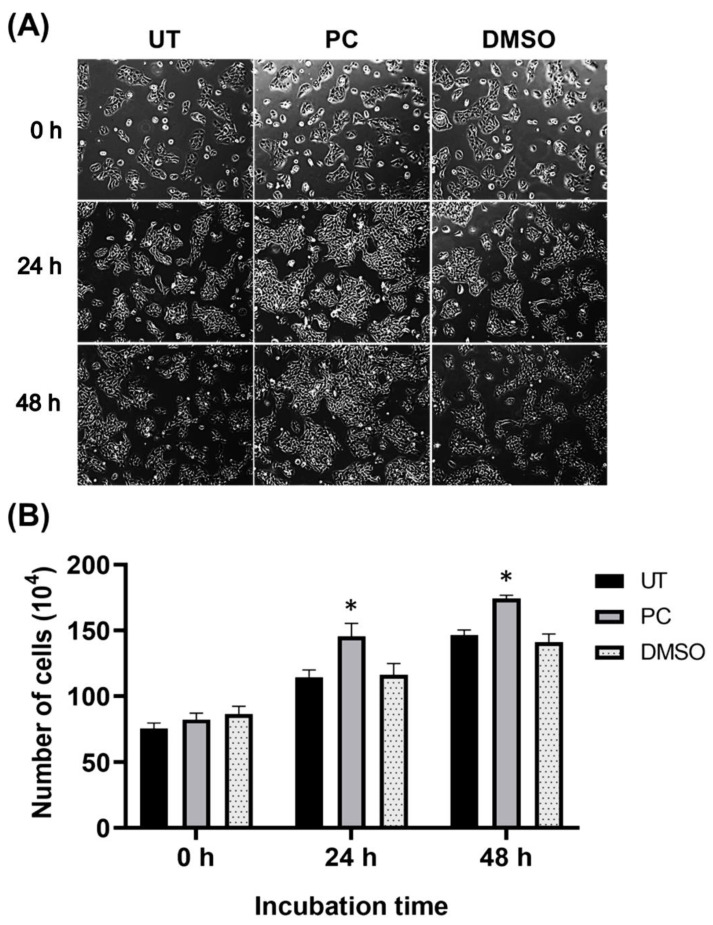
Effects of pinocembrin on human keratinocyte proliferation. (**A**) Phase-contrast observation of HaCaT cells treated with pinocembrin at 62.5 μM at 0, 24, and 48 h (10× magnification) compared with the untreated group and the DMSO control group; (**B**) Direct cell counting for number of human keratinocytes treated with pinocembrin at 62.5 μM and at 0, 24, and 48 h. Data from three experiments were analyzed and presented as mean ± SD. * *p* < 0.05 (compared to the untreated group, UT).

To explore the role of pinocembrin in activating human keratinocyte proliferation, we seeded HaCaT cells at low density, treated the cells with 62.5 μM pinocembrin, and visualized the changes in colony size over 48 h. As presented in Figure 3A, pinocembrin clearly stimulated an increase in colony size of HaCaT cells over the course of 48 h while the growing colony pattern of the DMSO control group was similar to that of the untreated group. To test the hypothesis that pinocembrin may stimulate keratinocyte proliferation, we directly counted the number of cells. Results demonstrated that at the 0 h time point, the number and distribution of the keratinocytes were seen to be similar to those of the control group and pinocembrin group. Obviously, pinocembrin at 62.5 μM significantly induced an increase in cell number of keratinocytes at 24 h and 48 h when compared with the untreated group and DMSO-treated group (Figure 3B).

### 2.4. Effects of Pinocembrin on the Signaling Pathways Regulating Keratinocyte Proliferation

**Figure 4:**
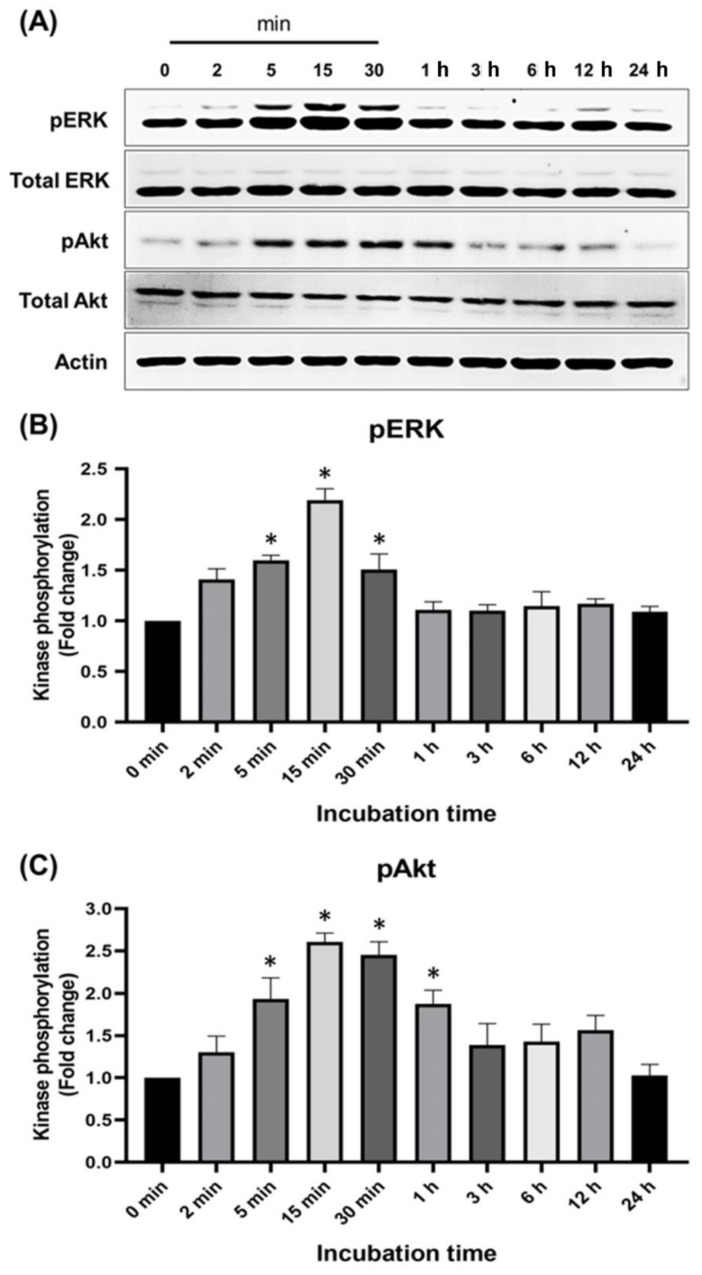
Effects of pinocembrin on MAPK and PI3K/Akt signaling pathways. (**A**) The activation status evaluated by the level of phosphorylation of ERK1/2 and Akt in the lysates of cells treated with 62.5 μM of pinocembrin at various time points; (**B**) Densitometric analysis of phosphorylated ERK1/2 in cells treated with pinocembrin at each time point; (**C**) Densitometric analysis of phosphorylated Akt in cells treated with pinocembrin at each time point. The total protein expression of each kinase was detected and used for normalization. Data from three experiments were analyzed and presented as mean ± SD. * *p* < 0.05 (compared to the 0 min group).

The MAPK and PI3K/Akt cascades are one of the most important cellular pathways for growth and survival of all type of cells including human keratinocytes. To inspect the effects of pinocembrin on these signaling cascades in human keratinocytes, the phosphorylation status of crucial cellular kinases (ERK1/2 and Akt) was monitored and measured upon treatment with pinocembrin at various time points (0–24 h). Results demonstrated that pinocembrin could rapidly activate phosphorylation of ERK1/2 protein within 2 min after pinocembrin addition. The signal intensity rapidly increased over time before it declined after 15 min (Figure 4A,B). The phosphorylation status of Akt was slightly delayed since a clear phosphorylation signal started to be seen 5 min post pinocembrin treatment, increased over time, and was stable for 1 h before it declined (Figure 4A,C). Noticeably, pinocembrin did not affect the expression of both ERK1/2 and Akt kinases. 

**Figure 5:**
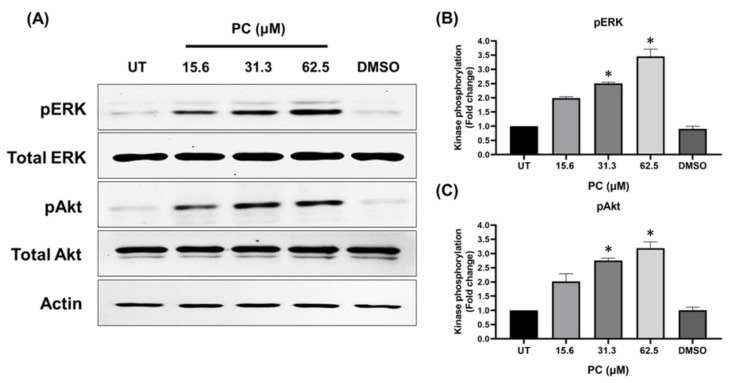
Concentration-dependent effects of pinocembrin on ERK1/2 and Akt activation. (**A**) Western blotting for ERK1/2 and Akt phosphorylation in HaCaT cells incubated with pinocembrin at 15.6, 31.3, or 62.5 μM for 15 min; (**B**) Densitometric analysis of phosphorylated ERK1/2 in lysates of cells treated with varied concentrations of pinocembrin; (**C**) Densitometric analysis of phosphorylated Akt in lysates of cells treated with varied concentrations of pinocembrin. Data from three experiments were analyzed and presented as mean ± SD. * *p* < 0.05 (compared to the control group, UT).

Moreover, we performed Western blotting to confirm the kinase-activating effects of pinocembrin at varied concentrations (15.6, 31.3, and 62.5 μM), and data clearly demonstrated that the phosphorylation form of the two kinases was significantly elevated when the dose of pinocembrin was increased without affecting the total protein level (Figure 5A). Quantification of immunoreactive signals demonstrated that after 15 min of pinocembrin treatment, phosphorylation of ERK1/2 and Akt was approximately 2-fold, 2.5- fold, and 3-fold for cells treated with pinocembrin at 15.6, 31.3, and 62.5 μM, respectively (Figure 5B,C).

**Figure 6:**
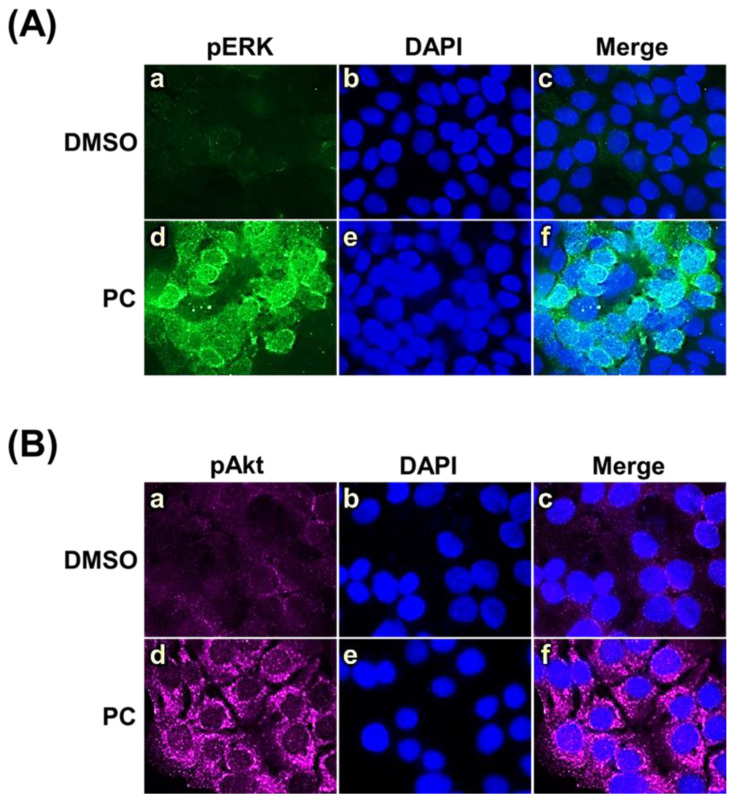
Immunofluorescence study determining ERK1/2 and Akt activation in cells treated with pinocembrin (62.5 μM). (**A**) Phosphorylated ERK1/2 (pERK1/2) (green); (**B**) Phosphorylated Akt (pAkt) (green). HaCaT cells were counterstained with DAPI to detect the nuclei (blue). Pictures were captured by a fluorescent microscope at 100× magnification.

Concomitantly, immunofluorescence analysis for the phosphorylation form of ERK1/2 (Figure 6A(d–f)) and Akt (Figure 6B(d–f)) definitively verified that pinocembrin rapidly induced the activation of these two kinases in individual cells compared to those cells treated with DMSO (Figure 6A(a–c),B(a–c)). 

### 2.5. Inhibitors That Specifically Inhibit MEK and PI3K Completely Block ERK1/2 and Akt Activation and HaCaT Cell Proliferation Induced by Pinocembrin

**Figure 7:**
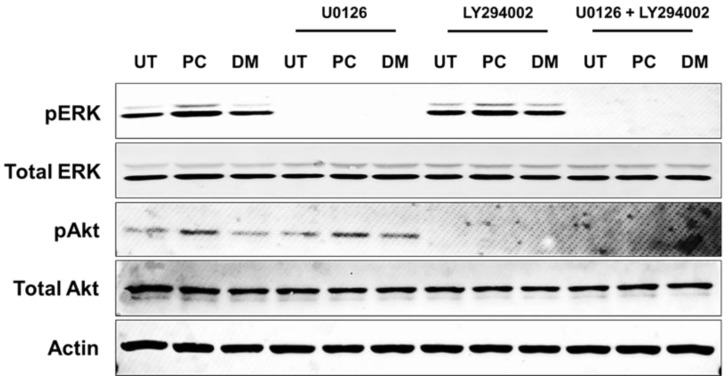
Western blot analysis detecting pERK1/2 and pAkt after incubation with pinocembrin at various concentrations (15.6, 31.3, and 62.5 μM) for 15 min in combination with U0126 and LY294002 which are specific inhibitors of MAPK and PI3K signaling. Data are obtained from three individual experiments.

Specific inhibitors which included U0126 (MEK inhibitor) and LY294002 (a PI3K inhibitor) were used to confirm whether pinocembrin specifically activates MAPK and PI3K transduction pathways. Western blot results confirmed that when U0126 was used, complete inhibition of ERK1/2 but not Akt was seen in keratinocytes treated with pinocembrin alone. Likewise, when LY294002 was applied, the phosphorylation of Akt but not ERK1/2 was blocked (Figure 7). Apparently, when both U0126 and LY294002 were applied to keratinocytes, no phosphorylation signal of ERK1/2 and Akt was detected (Figure 7). 

**Figure 8:**
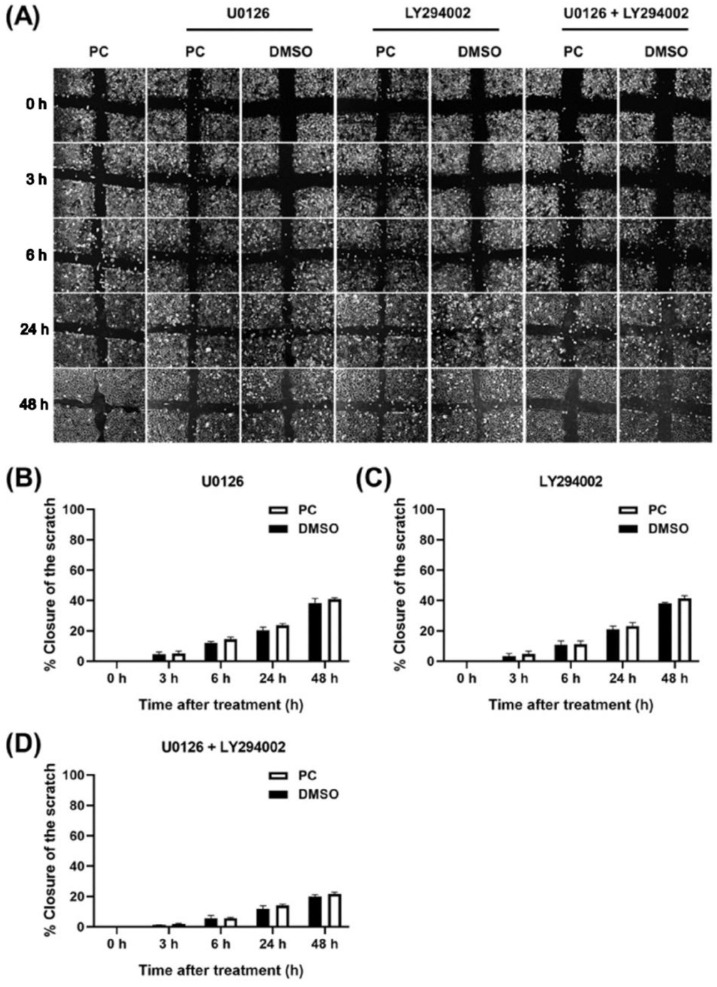
Scratch wound healing assay for investigating the monolayer wound closure accelerating effects of pinocembrin (62.5 μM) in the presence of U0126, LY294002, or combination of both U0126 and LY294002. (**A**) Phase-contrast microscopy (10× magnification) of scratch wound-healing assay with U0126, LY294002, or combination of U0126 and LY294002 over the course of 48 h. Percent closure of the scratch-wounded areas of human keratinocyte monolayer over the course of 48 h in the presence of (**B**) U0126, (**C**) LY294002, or (**D**) combination of both U0126 and LY294002. Data from three experiments were analyzed and presented as mean ± standard deviation.

Scratch wound healing was performed as a functional test to verify the results obtained from the experiments where the inhibitors were included. As anticipated, U0126 could suppress keratinocyte monolayer closure accelerating effects of PC over the course of 48 h. In addition, LY294002-treated group demonstrated a similar pattern of scratch healing inhibition. Doubtlessly, keratinocyte monolayer treated with pinocembrin with the presence of both U0126 and LY294002 exhibited the slowest rate of wound closure (Figure 8). These results show that pinocembrin induces keratinocyte proliferation mainly through activating MAPK and PI3K/Akt kinases.

## 3. Discussion

It is well known that the skin is an important organ that protects the internal viscera, prevents water loss, and protects against microorganisms and UV radiation. When an individual experiences open wound or skin damage, the sequential healing phases must occur for a specific duration at an optimal intensity to properly heal the wound [20]. In particular, wound healing is a complex process that requires the orchestration of different types of cells, cytokines, and growth factors in order to effectively close the wound [21]. Although the human body responds rapidly to reverse the functional integrity of skin, many negative factors may interfere with certain wound-healing phases causing delayed or improper tissue repair. One obvious factor is skin aging characterized by drying, roughness, atrophy, changes in pigmentation, wrinkling, and sagging which are caused by both intrinsic and extrinsic aging factors including exposure to ultraviolet irradiation [22]. Specifically, age-related delayed wound healing is linked to the aberration in the inflammation phase, and that includes slow infiltration of T-cell into the affected regions, changes in chemokine production, and reduced phagocytic activity of macrophages [23]. Additionally, angiogenesis, collagen synthesis, and re-epithelialization have been reported to be delayed in aged mice [24]. Besides an aspect of aging, other factors have been identified to be important factors that negatively influence wound healing. Those include oxygenation, infections, sex hormones, stress, diabetes, medications, obesity, alcohol consumption, smoking, and nutrition [25]. Improper or impaired wound healing caused by these factors may influence medical management and overall economy in every country. The United States has reported that the unmanaged skin trauma costs around 50 billion dollars, wound scarring from surgical treatments and trauma costs nearly 12 billion dollars, and burns cost 7.5 billion dollars per year [26]. Patients with complications, which are not responsive to currently available therapeutic agents, may experience delayed wound closure, and this enhances an increased risk of infections or development of certain new complications [27]. Therefore, the discovery of new wound healing-stimulating compounds would be clinically beneficial for the treatment of individuals who have a persistent skin wound with severe problems. 

Since ancient times, humans have relied on traditional herbal medicine for curing and preventing many diseases and conditions. Those medicinal plants have been revealed to exhibit strong biological activities which include anti-microbial, anti-inflammatory, and wound healing properties [28,29]. Currently, many studies have reported specific pharmacological effects of certain medicinal plants [30,31]. For these reasons, interest in medicinal plants and their active compounds is currently increasing. Pinocembrin is a type of flavonoid which is found in certain plants in the family of Piperaceae, Lauraceae, and Asteraceae which are mainly distributed in tropical and subtropical regions [13]. Besides, it is prevalent in different dietary sources such as Calabrian honey [32], licorice (Glycyrrhiza glabra) [33], and fingerroot (*Boesenbergia rotunda*) [19]. Many studies have reported about the anti-bacterial effects of pinocembrin [34,35]. About the wound healing-promoting properties, it was shown that *Boesenbergia rotunda* which contains pinocembrin [11] demonstrated cell proliferation-activating effects on human keratinocytes by promoting MAPK and PI3K/Akt signal transduction pathways [18]. 

Our current study focused on investigating the specific cellular signaling pathways at which pinocembrin acts on to promote proliferation and survival of human keratinocytes. Cell viability testing demonstrated that pinocembrin at a specific non-toxic range could drastically increase human keratinocyte viability. These results suggest that pinocembrin may elevate the rate of keratinocyte division. We further examined this hypothesis by focusing mainly on the influence of pinocembrin on cell proliferation and con-firmed that this compound truly stimulated an increase in cell number and enhanced the rate of monolayer wound healing. Moreover, Western blot analysis and immunofluorescence study clearly verified that MAPK/ERK and PI3K/Akt signal transduction pathway were rapidly stimulated in response to pinocembrin. When specific inhibitors of these two pathways were used, the kinase-stimulating effects of pinocembrin completely disappeared. Data from our findings indicate that pinocembrin rapidly stimulates functional kinases responsible for stimulating the growth (MAPK) and survival (PI3K/Akt) of human keratinocytes. Similar effects at the level of signal transduction pathway have been reported previously by our group where the ethanolic extract of *Boesenbergia rotunda* was examined [18], and pinocembrin has been identified by many studies as one of the major constituents of this plant [11,36]. It is well characterized that major mitogen-activated protein (MAP) kinases are key molecular players that stimulate the proliferation and differentiation of many cells, including keratinocytes where the effect is stimulated by growth factors, the level of intracellular calcium, and cytokines [37]. Furthermore, this signaling conveyed by the upstream kinase (MEKK1) induces the expression of responsible genes involved in wound re-epithelialization [38]. The PI3K/Akt signaling cascade also regulates cell proliferation, cell migration, and cell survival [39,40]. Interestingly, a previous study demonstrated that pinocembrin and its linolenoyl ester derivatives can promote wound healing in human keratinocytes through a GPR120/FFA4 mediated pathway [12]. Specifically, this study showed that pinocembrin was hybridized with fatty acids by using pancreatic porcine lipase, and its wound healing activity was mediated by GPR120/β-arrestin complexation. GPR120 is a G-protein-coupled receptor 120 that has been reported to crosstalk with the PI3K/Akt–NF-kB pathway [41]. Similarly, it has been explored that ERK1/2 is a functional kinase working downstream of GPR120 to modulate a series of biologic processes [42,43]. Nevertheless, how pinocembrin exactly interacts with certain molecular players remains to be identified. One way to obtain such information is through conducting a structural interaction assay as well as rational redesign of a functional protein kinase-substrate interaction [44] to probe the functional consequences of specific phosphorylation events in keratinocytes upon pinocembrin stimulation.

**Figure 9:**
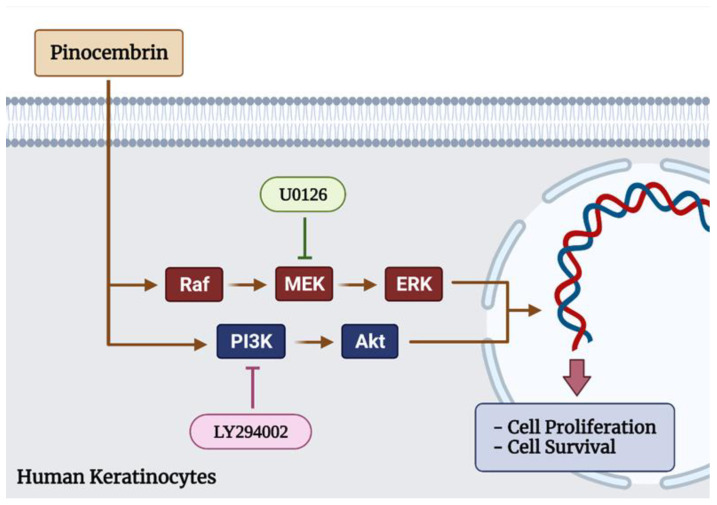
Schematic figure illustrating that pinocembrin regulates human keratinocyte proliferation and survival. Pinocembrin increases the proliferation and survival of keratinocytes through both MAPK signal transduction pathway and PI3K/Akt signaling pathway.

On the basis that keratinocytes in the epidermis play a crucial role on re-epithelialization which is a process for the restoration of the epidermis after injury, and on our recent discovery that pinocembrin is an active compound that rapidly targets certain cellular molecules resulting in the activation of the MAPK and PI3K/Akt (as illustrated in Figure 9), this sheds light on this natural compound for the possible development as an alternative agent required for the full regenerative function of keratinocytes, especially in patients who are less responsive to other treatments or patients with delayed wound healing. 

## 4. Materials and Methods

### 4.1. Pinocembrin Preparation

Pinocembrin was purchased from Sigma-Aldrich, St. Louis, MO, USA (Product Number: P5239-50 mg, Batch Number: MKCM4285). After purchase, Pinocembrin stock solution was prepared to be 195 mM in 100% DMSO, aliquoted, and stored at −20 °C. For each experiment, pinocembrin stock was directly diluted in cell culture media before each treatment.

### 4.2. Cell Lines and Cell Cultures

HaCaT keratinocytes were procured from CLS Cell Lines Service GmbH (Eppelheim, Baden-Wurttemberg, Germany). The cells were maintained in complete media, which was Dulbecco’s modified Eagle’s media (DMEM) (Gibco, New York, NY, USA), with the addition of 10% fetal bovine serum (Merck KGaA, Darmstadt, Germany), 100 U/mL penicillin, and 100 μg/mL streptomycin (both drugs from Thermo Fisher Scientific, Waltham, MA, USA). HaCaT cells were cultured in an incubator under a humidified atmosphere at 37 °C, 5% CO_2_. Media were changed every 2–3 days, and sub-culture was performed when the cells reached approximately 90% confluence. 

### 4.3. Cell Viability Assay

Following, 3-(4,5-dimethylthiazol-2-yl)-2,5-diphenyltetrazolium bromide (MTT) was used to evaluate the cell viability of pinocembrin-treated human keratinocytes. In 96-well plates (Thermo Fisher Scientific, USA), we seeded HaCaT cells at 2 × 10^5^ cells per well (200 µL per well) in complete media overnight. Next day, pinocembrin was diluted in FBS-free media to a working concentration of 500 µM, then the concentration was further diluted by 2-fold dilution to create a concentration range of 0.5–500 µM. The adhered cells were treated with these varied concentrations of pinocembrin for 48 h in FBS-free media. DMSO (a vehicle control) was diluted in a similar way to make a concentration range of 0.001–0.05% control. After 48 h of pinocembrin exposure, MTT reagent (stock 5 mg/mL) was added to each well (25 µL per well), and cells were incubated in a CO_2_ incubator for 1 h for formazan formation. After media were aspirated, formazan was dissolved with DMSO (µL per well). The difference in color intensity was determined by a microplate reader set at 570 nm. 

### 4.4. Phase-Contrast Microscopy 

Phase-contrast observation was performed to monitor keratinocyte colony expansion over time upon pinocembrin treatment. Human keratinocytes were seeded at 0.1 × 10^6^ cells in 24-well plates in complete media overnight. Then, media were replaced to be FBS-free DMEM, and cells were treated with pinocembrin at 62.5 μM or DMSO. The micrograph of pinocembrin-treated human keratinocytes at various time points (0, 24, and 48 h) were captured with 10× magnification by using Axio Vert.A1 microscope (Carl Zeiss Suzhou Co., Ltd., Suzhou, China)

### 4.5. Direct Measurement of Cell Number 

HaCaT cells were directly counted for measuring the changes in cell amount in different treating conditions. Human keratinocytes were seeded at 0.1 × 10^6^ cells in 24-well plates in DMEM with FBS and incubated overnight. Adherent cells were then treated with 62.5 μM pinocembrin in media without FBS. The total number of cells were obtained at 0, 24, and 48 h by using CellDrop™ Automated Cell Counters (DeNovix Inc., Wilmington, DE, USA).

### 4.6. Cellular Wound-Healing Activity Assay

Human keratinocyte monolayer wound-healing assay was performed to investigate wound closure-accelerating effects of pinocembrin. The seeded HaCaT cells (0.02 × 10^6^ cells) were allowed to attain confluence in complete media in 24-well plates. A scratch wound in a vertical and horizontal crossline fashion were created by using SPLScar™ Scratcher (SPL Life Sciences, Gyeonggi-do, Korea). Each well was washed one time with sterile phosphate buffer saline (PBS), and the cells were then treated with pinocembrin at 62.5 μM or DMSO in DMEM with no FBS. The changes in the wounded areas were observed and photographed at 0 h, 3 h, 6 h, 24 h, and 48 h using a phase-contrast Axio Vert.A1 microscope, with 10× magnification. In some experiments, a MEK-1inhibitor (U0126) and a PI3K inhibitor (LY294002) were included to confirm the molecular mechanism of pinocembrin at the signaling level. 

### 4.7. Western Blot Analysis

To investigate the possible mode of action of pinocembrin on cell proliferation signaling pathways and cell survival signaling pathways, we detected the phosphorylated forms of ERK1/2 kinases and protein kinase B (AKT) Western blotting as described in previous studies [18]. Human keratinocytes were seeded at 0.2 × 10^6^ cells in 24-well plates overnight. The cells were then incubated with pinocembrin at various doses (15.6, 31.3, and 62.5 μM) for 15 min or treated with 62.5 μM at different time points (0 min, 2 min, 5 min, 15 min, 30 min, 1 h, 3 h, 6 h, 12 h, and 24 h). In some experiments, cells were exposed to U0126 or LY294002 for 2 h prior to treatment with pinocembrin or DMSO. Cell lysates were prepared with 1X reducing Laemmli buffer (150 μL), heated at 95 °C for 5 min in a heat box, and subjected to electrophoresis (SDS-PAGE) and electroblotting. Target proteins on the membranes were detected with specific antibodies (Cell Signaling Technology, Boston, MA, USA) which included antibodies against ERK1/2 (catalog no.9107), phosphorylated ERK1/2 (catalog no.4370), Akt (catalog no.2920), phosphorylated Akt (catalog no.4060), and actin (catalog no.3700) at 4 °C overnight, with gentle agitation. All antibodies were diluted as instructed by the company. After three washes with TBS-T, membranes were incubated with 1:10,000 of anti-mouse IgG-IRDye^®^800CW (catalog no.926–32210) or 1:10,000 of anti-rabbit IgG-IRDye^®^680RT (catalog no.926–68071) (LI−COR Biosciences, USA) for 2 h at room temperature. The signal of targeted protein on membranes were detected with an Odyssey^®^ CLx Imaging System (LI−COR Biosciences, Lincoln, NE, USA). Densitometric analysis of each protein band was performed by using ImageJ software.

### 4.8. Immunofluorescence Study

To stain ERK and Akt phosphorylation signal in keratinocyte treated with pinocembrin, we performed immunostaining using the same primary antibodies as above. Human keratinocytes were seeded at 0.1 × 10^6^ cells onto the sterile glass cover slips placed in 3 cm cell culture dishes overnight in complete media (DMEM). Cells were serum-starved for 24 h prior to treatment with 62.5 μM of pinocembrin for 15 min. Cells were then fixed with 4% paraformaldehyde or −20 °C absolute methanol (depending on the instruction of the company) for 15 min at room temperature. After three washes with PBS (each time for 5 min), paraformaldehyde-fixed cells were permeabilized with Triton X-100 (0.3% in sterile PBS) for 5 min at room temperature. Sample coverslips were incubated with 1% bovine serum albumin (diluted in PBS-T) for 1 h at room temperature to block non-specific binding. By following the instruction from the company, sample coverslips were incubated with certain primary antibodies at 4 °C overnight. All primary antibodies were the same ones used in Western blot analysis. After washing for three times with PBS, sample coverslips were incubated with anti-rabbit IgG-Alexa488 and 5 μg/mL of DAPI (nuclear staining) for 2 h at room temperature, in a light-protecting container. Sample coverslips were washed three times with PBS and one time with distilled water, and mounted with Fluoromount-G (SouthernBiotech, Birmingham, AL, USA). The fluorescent signal of the targeted proteins was visualized at 100× magnification and captured with Axio Vert.A1 microscope (Carl Zeiss Suzhou Co., Ltd., Suzhou, China), equipped with the Zen 2.6 (blue edition) Software for the Zeiss Axiocam 506 color microscope camera.

### 4.9. Statistical Analysis

Data from each experiment were obtained from at least three independent replicates, and presented as mean ± SD. One-way analysis of variance (ANOVA) with Tukey’s post hoc multiple comparisons on raw data reads by using GraphPad Prism 8.0.1 (GraphPad Software Inc., San Diego, CA, USA) was applied. A *p*-value less than 0.05 indicates statis-tical significance.

## 5. Conclusions

We revealed that pinocembrin is an active compound with strong stimulating effects on human keratinocytes. Pinocembrin positively regulates growth and survival of the cells through activating ERK1/2 and Akt. The ability of pinocembrin in modulating the function of crucial signaling kinases at their post-translational modification level highlights its distinct regenerative properties. This evidence supports the use of pinocembrin-containing plants and natural products as an alternative means for skin regeneration and wound healing when some patients fail the currently available treatments, or when the cost of certain wound-healing agents, such as growth factors, is unaffordable.

## Data Availability

Data is contained within the article.
